# 4-Hy­droxy­anilinium 2-chloro­acetate

**DOI:** 10.1107/S1600536812021411

**Published:** 2012-05-26

**Authors:** Ying-Chun Wang

**Affiliations:** a College of Chemistry and Chemical Engineering, Southeast University, Nanjing 210096, People’s Republic of China

## Abstract

In the crystal of the title salt, C_6_H_8_NO^+^·C_2_H_2_ClO_2_^−^, the 4-hy­droxy­anilinium cation links to adjacent chloro­acetate anions *via* N—H⋯O and O—H⋯O hydrogen bonds; weak C—H⋯O inter­actions also occur between the anions and cations.

## Related literature

For the structures and properties of related compounds, see: Chen *et al.* (2001 ▶); Wang *et al.* (2002 ▶); Xue *et al.* (2002 ▶); Huang *et al.* (1999 ▶); Zhang *et al.* (2001 ▶); Ye *et al.* (2008 ▶).
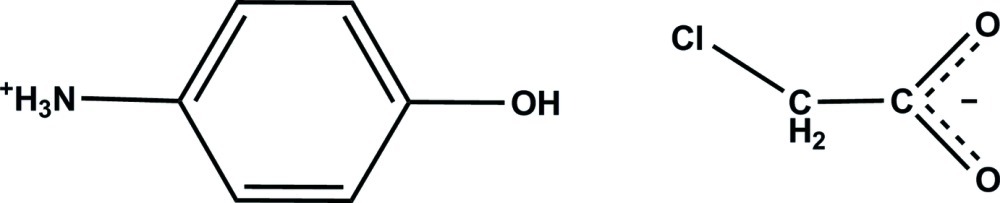


## Experimental

### 

#### Crystal data

C_6_H_8_NO^+^·C_2_H_2_ClO_2_^−^
*M* *_r_* = 203.62
Monoclinic, 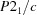

*a* = 10.702 (3) Å
*b* = 4.5242 (10) Å
*c* = 19.357 (7) Å
β = 96.825 (2)°
*V* = 930.6 (5) Å^3^
*Z* = 4
Mo *K*α radiation
μ = 0.38 mm^−1^
*T* = 173 K
0.10 × 0.05 × 0.05 mm

#### Data collection

Rigaku Mercury2 diffractometer
Absorption correction: multi-scan (*CrystalClear*; Rigaku, 2005 ▶) *T*_min_ = 0.910, *T*_max_ = 1.000
6248 measured reflections
2122 independent reflections
1699 reflections with *I* > 2σ(*I*)
*R*_int_ = 0.045

#### Refinement

*R*[*F* ^2^ > 2σ(*F* ^2^)] = 0.048
*wR*(*F* ^2^) = 0.104
*S* = 1.09
2122 reflections
119 parameters
4 restraints
H-atom parameters constrained
Δρ_max_ = 0.31 e Å^−3^
Δρ_min_ = −0.31 e Å^−3^

### 

Data collection: *CrystalClear* (Rigaku, 2005 ▶); cell refinement: *CrystalClear*; data reduction: *CrystalClear*; program(s) used to solve structure: *SHELXS97* (Sheldrick, 2008 ▶); program(s) used to refine structure: *SHELXL97* (Sheldrick, 2008 ▶); molecular graphics: *SHELXTL* (Sheldrick, 2008 ▶); software used to prepare material for publication: *SHELXTL*.

## Supplementary Material

Crystal structure: contains datablock(s) I, global. DOI: 10.1107/S1600536812021411/xu5534sup1.cif

Structure factors: contains datablock(s) I. DOI: 10.1107/S1600536812021411/xu5534Isup2.hkl

Supplementary material file. DOI: 10.1107/S1600536812021411/xu5534Isup3.cml

Additional supplementary materials:  crystallographic information; 3D view; checkCIF report

## Figures and Tables

**Table 1:** Hydrogen-bond geometry (Å, °)

*D*—H⋯*A*	*D*—H	H⋯*A*	*D*⋯*A*	*D*—H⋯*A*
N1—H1*A*⋯O3	0.91	1.86	2.755 (3)	166
N1—H1*B*⋯O2^i^	0.91	1.87	2.777 (3)	171
N1—H1*C*⋯O2^ii^	0.91	1.90	2.762 (2)	157
O1—H1⋯O3^iii^	0.82	1.87	2.683 (2)	172
C8—H8*A*⋯O1^iv^	0.99	2.38	3.319 (3)	159

Symmetry codes: (i) 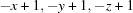; (ii) 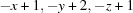; (iii) 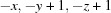; (iv) 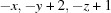.

**Fig. 1.**
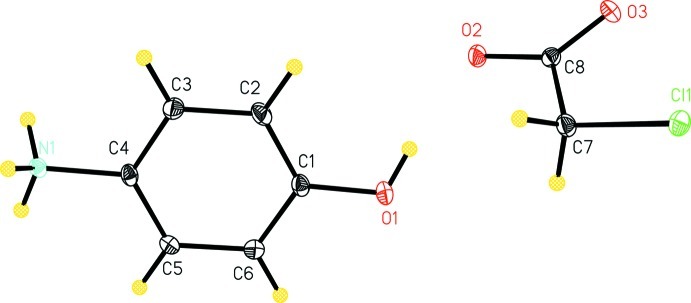
A view of the asymmetric unit with the atomic numbering scheme. The displacement ellipsoids were drawn at the 30% probability level.

## supplementary crystallographic information

### Comment

Simple organic salts containing strong intrermolecular H-bonds have attracted an attention as materials which display ferroelectric-paraelectric phase transitions (Chen *et al.*, 2001; Huang *et al.*, 1999; Zhang *et al.*, 2001). With the purpose of obtaining phase transition crystals of organic salts, various organic molecules have been studied and a series of new crystal materials have been elaborated (Wang *et al.*, 2002; Xue *et al.*, 2002; Ye *et al.*, 2008). Herewith, we present the synthesis and crystal structure of the title compound, 4-hydroxyanilinium 2-chloroacetate.

In the title compound (Fig. 1), the bond lengths and angles have normal values. The asymmetric unit was composed of one 4-hydroxyanilinium cation and one 2-chloroacetate anion. The protonated N atom was involved in strong intramolecular N—H···O hydrogen bonds with the N···O distances of 2.777 (3)Å, 2.762 (2)Å and 2.755 (3)Å, respectively. The N—H···O and O—H···O H-bonding interactions connected the ion units into a 2D network parallel to the *ab*-plane. The weak intermolecular C8—H8A···O1 interaction was presented in the crystal structure with C8···O1 = 3.319 (3)Å.

### Experimental

The 2-chloroacetic acid (10 mmol), 4-aminophenol (10 mmol) and ethanol (50 mL) were added into a 100 mL flask. The mixture was stirred at 333 K for 2 h, and then the precipitate was filtered out. Colourless crystals suitable for X-ray diffraction were obtained by slow evaporation of the solution.

### Refinement

All the H atoms attached to C atoms were situated into the idealized positions and treated as riding with C–H = 0.95 Å (aromatic) and C–H = 0.99 Å (methylene) with *U_iso_*(H) = 1.2*U_eq_*(C). The positional parameters of the H atoms bonded to N and O were located in a difference Fourier map and refined with restraints of H—N = 0.91 (2) and H—O = 0.82 (2) Å, *U_iso_*(H) = 1.5*U_eq_*(N,O).

### Crystal data

**Table:**

C_6_H_8_NO^+^·C_2_H_2_ClO_2_^−^	*F*(000) = 424
*M**_r_* = 203.62	*D*_x_ = 1.453 Mg m^−^^3^
Monoclinic, *P*2_1_/*c*	Mo *K*α radiation, λ = 0.71073 Å
Hall symbol: -P 2ybc	Cell parameters from 2122 reflections
*a* = 10.702 (3) Å	θ = 2.7–27.5°
*b* = 4.5242 (10) Å	µ = 0.38 mm^−^^1^
*c* = 19.357 (7) Å	*T* = 173 K
β = 96.825 (2)°	Block, colorless
*V* = 930.6 (5) Å^3^	0.10 × 0.05 × 0.05 mm
*Z* = 4	

### Data collection

**Table:**

Rigaku Mercury2 diffractometer	2122 independent reflections
Radiation source: fine-focus sealed tube	1699 reflections with *I* > 2σ(*I*)
Graphite monochromator	*R*_int_ = 0.045
Detector resolution: 13.6612 pixels mm^-1^	θ_max_ = 27.5°, θ_min_ = 2.7°
CCD profile fitting scans	*h* = −12→13
Absorption correction: multi-scan (*CrystalClear*; Rigaku, 2005)	*k* = −5→5
*T*_min_ = 0.910, *T*_max_ = 1.000	*l* = −25→23
6248 measured reflections	

### Refinement

**Table:**

Refinement on *F*^2^	Primary atom site location: structure-invariant direct methods
Least-squares matrix: full	Secondary atom site location: difference Fourier map
*R*[*F*^2^ > 2σ(*F*^2^)] = 0.048	Hydrogen site location: inferred from neighbouring sites
*wR*(*F*^2^) = 0.104	H-atom parameters constrained
*S* = 1.09	*w* = 1/[σ^2^(*F*_o_^2^) + (0.041*P*)^2^ + 0.4*P*] where *P* = (*F*_o_^2^ + 2*F*_c_^2^)/3
2122 reflections	(Δ/σ)_max_ < 0.001
119 parameters	Δρ_max_ = 0.31 e Å^−^^3^
4 restraints	Δρ_min_ = −0.31 e Å^−^^3^

### Special details

**Table:**

Geometry. All esds (except the esd in the dihedral angle between two l.s. planes) are estimated using the full covariance matrix. The cell esds are taken into account individually in the estimation of esds in distances, angles and torsion angles; correlations between esds in cell parameters are only used when they are defined by crystal symmetry. An approximate (isotropic) treatment of cell esds is used for estimating esds involving l.s. planes.
Refinement. Refinement of F^2^ against ALL reflections. The weighted R-factor wR and goodness of fit S are based on F^2^, conventional R-factors R are based on F, with F set to zero for negative F^2^. The threshold expression of F^2^ > 2sigma(F^2^) is used only for calculating R-factors(gt) etc. and is not relevant to the choice of reflections for refinement. R-factors based on F^2^ are statistically about twice as large as those based on F, and R- factors based on ALL data will be even larger.

### Fractional atomic coordinates and isotropic or equivalent isotropic displacement parameters (Å^2^)

**Table:**

	*x*	*y*	*z*	*U*_iso_*/*U*_eq_	
Cl1	0.41622 (5)	1.32273 (13)	0.29181 (3)	0.02016 (16)	
O3	0.29262 (14)	0.7514 (4)	0.42037 (8)	0.0222 (4)	
O2	0.48355 (14)	0.9420 (4)	0.41518 (8)	0.0188 (4)	
N1	0.37140 (16)	0.5601 (4)	0.55354 (10)	0.0163 (4)	
H1A	0.3555	0.6038	0.5074	0.024*	
H1B	0.4170	0.3902	0.5590	0.024*	
H1C	0.4158	0.7104	0.5760	0.024*	
O1	−0.08865 (14)	0.4356 (4)	0.66202 (9)	0.0250 (4)	
H1	−0.1461	0.3684	0.6347	0.037*	
C2	0.1616 (2)	0.3348 (6)	0.54972 (13)	0.0223 (5)	
H2A	0.1781	0.2289	0.5094	0.027*	
C7	0.3694 (2)	0.9122 (5)	0.39246 (11)	0.0163 (5)	
C1	0.2513 (2)	0.5212 (5)	0.58293 (12)	0.0164 (5)	
C6	0.2298 (2)	0.6739 (6)	0.64231 (12)	0.0204 (5)	
H6A	0.2929	0.7995	0.6651	0.025*	
C4	0.0232 (2)	0.4565 (5)	0.63473 (12)	0.0184 (5)	
C5	0.1148 (2)	0.6413 (6)	0.66814 (12)	0.0235 (5)	
H5A	0.0989	0.7458	0.7088	0.028*	
C3	0.0466 (2)	0.3032 (6)	0.57581 (13)	0.0231 (5)	
H3A	−0.0159	0.1760	0.5531	0.028*	
C8	0.3133 (2)	1.0670 (5)	0.32664 (12)	0.0191 (5)	
H8A	0.2366	1.1738	0.3364	0.023*	
H8B	0.2873	0.9156	0.2909	0.023*	

### Atomic displacement parameters (Å^2^)

**Table:**

	*U*^11^	*U*^22^	*U*^33^	*U*^12^	*U*^13^	*U*^23^
Cl1	0.0217 (3)	0.0185 (3)	0.0211 (3)	−0.0005 (2)	0.0056 (2)	0.0027 (2)
O3	0.0177 (8)	0.0311 (10)	0.0180 (8)	−0.0054 (7)	0.0023 (7)	0.0060 (7)
O2	0.0146 (8)	0.0172 (8)	0.0236 (9)	−0.0023 (7)	−0.0018 (6)	0.0011 (7)
N1	0.0141 (9)	0.0171 (10)	0.0177 (10)	−0.0021 (8)	0.0020 (7)	0.0020 (8)
O1	0.0135 (8)	0.0374 (11)	0.0245 (9)	−0.0036 (8)	0.0041 (7)	0.0002 (8)
C2	0.0221 (12)	0.0226 (12)	0.0232 (12)	−0.0053 (11)	0.0066 (10)	−0.0074 (10)
C7	0.0161 (11)	0.0166 (11)	0.0165 (11)	0.0005 (9)	0.0035 (9)	−0.0015 (9)
C1	0.0126 (10)	0.0173 (12)	0.0193 (12)	−0.0005 (9)	0.0023 (9)	0.0059 (9)
C6	0.0158 (11)	0.0267 (13)	0.0181 (11)	−0.0025 (10)	−0.0012 (9)	−0.0015 (10)
C4	0.0135 (10)	0.0217 (12)	0.0197 (12)	0.0005 (9)	0.0012 (9)	0.0069 (10)
C5	0.0191 (11)	0.0338 (15)	0.0175 (12)	−0.0009 (11)	0.0014 (9)	−0.0015 (10)
C3	0.0183 (11)	0.0225 (12)	0.0285 (13)	−0.0089 (10)	0.0027 (10)	−0.0057 (11)
C8	0.0169 (11)	0.0197 (12)	0.0205 (12)	−0.0036 (10)	0.0012 (9)	0.0031 (10)

### Geometric parameters (Å, º)

**Table:**

Cl1—C8	1.784 (2)	C2—H2A	0.9500
O3—C7	1.265 (3)	C7—C8	1.514 (3)
O2—C7	1.256 (3)	C1—C6	1.384 (3)
N1—C1	1.477 (3)	C6—C5	1.390 (3)
N1—H1A	0.9100	C6—H6A	0.9500
N1—H1B	0.9100	C4—C3	1.383 (3)
N1—H1C	0.9100	C4—C5	1.389 (3)
O1—C4	1.368 (3)	C5—H5A	0.9500
O1—H1	0.8203	C3—H3A	0.9500
C2—C1	1.378 (3)	C8—H8A	0.9900
C2—C3	1.392 (3)	C8—H8B	0.9900
			
C1—N1—H1A	109.5	C1—C6—H6A	120.4
C1—N1—H1B	109.5	C5—C6—H6A	120.4
H1A—N1—H1B	109.5	O1—C4—C3	122.4 (2)
C1—N1—H1C	109.5	O1—C4—C5	117.6 (2)
H1A—N1—H1C	109.5	C3—C4—C5	119.9 (2)
H1B—N1—H1C	109.5	C4—C5—C6	120.2 (2)
C4—O1—H1	113.6	C4—C5—H5A	119.9
C1—C2—C3	119.4 (2)	C6—C5—H5A	119.9
C1—C2—H2A	120.3	C4—C3—C2	120.1 (2)
C3—C2—H2A	120.3	C4—C3—H3A	119.9
O2—C7—O3	124.5 (2)	C2—C3—H3A	119.9
O2—C7—C8	121.14 (19)	C7—C8—Cl1	114.65 (16)
O3—C7—C8	114.38 (19)	C7—C8—H8A	108.6
C2—C1—C6	121.2 (2)	Cl1—C8—H8A	108.6
C2—C1—N1	118.9 (2)	C7—C8—H8B	108.6
C6—C1—N1	119.9 (2)	Cl1—C8—H8B	108.6
C1—C6—C5	119.2 (2)	H8A—C8—H8B	107.6
			
C3—C2—C1—C6	1.0 (4)	C1—C6—C5—C4	0.3 (4)
C3—C2—C1—N1	−178.1 (2)	O1—C4—C3—C2	179.3 (2)
C2—C1—C6—C5	−1.0 (4)	C5—C4—C3—C2	−0.3 (4)
N1—C1—C6—C5	178.2 (2)	C1—C2—C3—C4	−0.4 (4)
O1—C4—C5—C6	−179.3 (2)	O2—C7—C8—Cl1	−7.3 (3)
C3—C4—C5—C6	0.3 (4)	O3—C7—C8—Cl1	173.97 (17)

### Hydrogen-bond geometry (Å, º)

**Table:**

*D*—H···*A*	*D*—H	H···*A*	*D*···*A*	*D*—H···*A*
N1—H1*A*···O3	0.91	1.86	2.755 (3)	166
N1—H1*B*···O2^i^	0.91	1.87	2.777 (3)	171
N1—H1*C*···O2^ii^	0.91	1.90	2.762 (2)	157
O1—H1···O3^iii^	0.82	1.87	2.683 (2)	172
C8—H8*A*···O1^iv^	0.99	2.38	3.319 (3)	159

Symmetry codes: (i) −*x*+1, −*y*+1, −*z*+1; (ii) −*x*+1, −*y*+2, −*z*+1; (iii) −*x*, −*y*+1, −*z*+1; (iv) −*x*, −*y*+2, −*z*+1.
